# Supplementary material for: Epidemiology of thyroid disorders in the Lifelines Cohort Study (the Netherlands)
Source: PLoS One. 2020 Nov 25;15(11):e0242795. doi: 10.1371/journal.pone.0242795 (PMC7688129; doi:10.1371/journal.pone.0242795)
Supplement: S1 Table — (DOCX) [file pone.0242795.s001.docx]

**S1 Table. Baseline characteristics of the 152180 participants.**

|  | No thyroid hormone | Thyroid hormone users | P-value |
| --- | --- | --- | --- |
| Number | 147390 | 4672 |  |
| Men / women | 62563 / 84827 | 550 / 4122 |  |
| % women | 57.6 | 88.2 | <0.001 |
| Age (years) | 44.4 ± 13.1 | 50.8 ± 12.8 | <0.001 |
| BMI (kg/m^2^) | 26.0 ± 4.3 | 27.6 ± 5.4 | <0.001 |
| Waist (cm) | 90 ± 12 | 92 ± 14 | <0.001 |
| Waist-hip ratio | 0.91 ± 0.09 | 0.89 ± 0.08 | <0.001 |
| BMI >30 kg/m^2^ (%) | 15.4 | 26.5 | <0.001 |
| Systolic BP (mmHg) | 125 ± 15 | 126 ± 16 | 0.144 |
| Diastolic BP (mmHg) | 74 ± 9 | 73 ± 9 | <0.001 |
| Heart rate (b/min) | 71 ± 11 | 71 ± 11 | 0.516 |
| Creatinine (mcmol/L) | 74 ± 14 | 70 ± 14 | <0.001 |
| Fasting glucose (mmol/L) | 5.0 ± 0.8 | 5.1 ± 1.1 | <0.001 |
| HbA1c (%) | 5.5 ± 0.4 | 5.7 ± 0.6 | <0.001 |
| Total cholesterol (mmol/L) | 5.06 ± 1.00 | 5.21 ± 1.04 | <0.001 |
| HDL-cholesterol (mmol/L) | 1.49 ± 0.39 | 1.53 ± 0.40 | <0.001 |
| LDL-cholesterol (mmol/L) | 3.22 ± 0.92 | 3.30 ± 0.94 | <0.001 |
| Triglycerides (mmol/L) | 0.98 (0.71-1.40) | 1.03 (0.75-1.45) | <0.001 |
| % with metabolic syndrome | 13.0 | 18.3 | <0.001 |
| % with type 2 diabetes | 3.2 | 6.5 | <0.001 |
| % using statin | 6.0 | 13.3 | <0.001 |
| % using BP-lowering drug(s) | 12.1 | 24.6 | <0.001 |
| % using amiodarone | 0.03 | 0.2 | <0.001 |
| Total number of medications | 1.2 (0-2) | 3.2 (1-4) | <0.001 |

Data are given as mean ± SD, median (IQR), absolute number, or percentage.

Participants reporting the use of thyroid blockers (methimazole or propylthiouracil) were excluded.
