# Supplementary material for: Epidemiology of thyroid disorders in the Lifelines Cohort Study (the Netherlands)
Source: PLoS One. 2020 Nov 25;15(11):e0242795. doi: 10.1371/journal.pone.0242795 (PMC7688129; doi:10.1371/journal.pone.0242795)
Supplement: S2 Table — (DOCX) [file pone.0242795.s002.docx]

**S2 Table. Thyroid hormone levels measured at baseline in 39859 Lifelines participants*.**

|  | Use of levothyroxine | | |
| --- | --- | --- | --- |
|  | No | Yes | P-value |
| Number | 38736 | 1123 |  |
| TSH (mIU/L) | 2.12 (<0.001 - 535) | 2.36 (<0.001 - 146) | 0.037 |
| FT4 (pmol/L) | 15.7 ± 2.1 | 18.3 ± 3.6 | <0.001 |
| FT3 (pmol/L) | 5.25 ± 0.73 | 4.59 ± 0.74 | <0.001 |

* Participants reporting the use of thyroid blockers (methimazole or propylthiouracil), liothyronine, animal source thyroid hormone and amiodarone were excluded.

Data are given as mean ± SD, or median (range).
