# Supplementary material for: Epidemiology of thyroid disorders in the Lifelines Cohort Study (the Netherlands)
Source: PLoS One. 2020 Nov 25;15(11):e0242795. doi: 10.1371/journal.pone.0242795 (PMC7688129; doi:10.1371/journal.pone.0242795)
Supplement: S3 Table — (DOCX) [file pone.0242795.s003.docx]

**S3 Table. Distribution of TSH class per age group (in %) in NHANES participants not using thyroid hormone medication at baseline.**

|  |  | TSH levels (mIU/L) | | | |
| --- | --- | --- | --- | --- | --- |
| Age group (years) | N | <0.4 | 0.4-4.0 | 4.01-10.0 | ≥10.0 |
| 18-29 | 1816 | 2.5 | 94.3 | 2.7 | 0.5 |
| 30-39 | 1479 | 2.6 | 92.6 | 4.2 | 0.6 |
| 40-49 | 1392 | 1.8 | 94.1 | 4.0 | 0.1 |
| 50-59 | 1293 | 1.8 | 92.6 | 4.9 | 0.7 |
| 60-69 | 1308 | 2.3 | 91.4 | 5.8 | 0.5 |
| 70-79 | 869 | 1.4 | 90.2 | 7.8 | 0.6 |
| ≥80 | 448 | 2.7 | 86.6 | 9.8 | 0.9 |
| Total n (%) | 8605 | 186 (2.1) | 7957 (92.5) | 419 (4.9) | 43 (0.5) |
