# Supplementary material for: Epidemiology of thyroid disorders in the Lifelines Cohort Study (the Netherlands)
Source: PLoS One. 2020 Nov 25;15(11):e0242795. doi: 10.1371/journal.pone.0242795 (PMC7688129; doi:10.1371/journal.pone.0242795)
Supplement: S4 Table — (DOCX) [file pone.0242795.s004.docx]

**S4 Table. Self-reported thyroid disorders or previous surgery using open questions at baseline.**

| HEALTH73: another disorder that you have not mentioned yet? | |
| --- | --- |
| Hypothyroidism | 152 |
| Hyperthyroidism not specified | 16 |
| Graves’ disease | 33 |
| Nodular / goiter | 18 |
| Thyroid cancer | 12 |
| Thyroiditis | <10 |
| Thyroid disorder, not specified | 43 |

| HEALTH74: previous thyroid surgery | |
| --- | --- |
| Unspecified reason | 371 |
| Nodular / goiter | 163 |
| Cyst of the thyroid | 66 |
| Thyroid cancer | 43 |
| Thyrotoxicosis | 22 |
| Graves’ disease | <10 |
| No thyroid surgery | 151506 |
