# Supplementary material for: Epidemiology of thyroid disorders in the Lifelines Cohort Study (the Netherlands)
Source: PLoS One. 2020 Nov 25;15(11):e0242795. doi: 10.1371/journal.pone.0242795 (PMC7688129; doi:10.1371/journal.pone.0242795)
Supplement: S5 Table — (DOCX) [file pone.0242795.s005.docx]

**S5 Table. Self-reported thyroid disorders using open questions asking for other disorders during the follow-up questionnaires.**

HEALTH100: Another disorder that you have not mentioned yet?

|  | Entire cohort | Those with thyroid hormone measurement |
| --- | --- | --- |
| N | 136776 | 37741 |
| Hypothyroidism | 778 | 236 |
| Hyperthyroidism not specified | 143 | 49 |
| Graves’ disease | 166 | 41 |
| Nodular / goiter | 168 | 43 |
| Thyroid cancer | <10 | <10 |
| Thyroiditis | 21 | <10 |
| Thyroid disorder, not specified | 317 | 97 |
