# Supplementary material for: Epidemiology of thyroid disorders in the Lifelines Cohort Study (the Netherlands)
Source: PLoS One. 2020 Nov 25;15(11):e0242795. doi: 10.1371/journal.pone.0242795 (PMC7688129; doi:10.1371/journal.pone.0242795)
Supplement: Attachment — Submitted filename: Response to Reviewers.docx [file pone.0242795.s006.docx]

P.O. Box 30 001, 9700 RB Groningen, the Netherlands

**Internal Medicine**

Head of department	Prof. dr. R.O.B. Gans

Division	Endocrinology

Head of Division	Prof. dr. B.H.R. Wolffenbuttel

To:

PLOS ONE
Prof. J. Heber and Dr. Naitza
Editor-in-Chief and Academic Editor

Date: August 14, 2020

Phone	0031 50 361 3962
E-mail	bwo@umcg.nl

Dear Professor Heber and Dr. Naitza,

We would like to thank you for your decision dated 27-07-2020 and the opportunity to resubmit a revised version of our manuscript entitled ‘Epidemiology of thyroid disorders in the Lifelines Cohort Study’ (PONE-D-20-13620). We would also like to thank the reviewers for their careful review and constructive comments on our manuscript.

In the revised manuscript, we have considered the comments and suggestions outlined by the Academic Editor and reviewers and have modified the manuscript accordingly. The comments are outlined below, followed by our response in a point-by-point manner. We also specified the data sharing restrictions. The changes made in the manuscript are as requested highlighted in the manuscript using Track Changes.

We believe the reviewers’ comments and subsequent revisions further strengthened our work and considerably improved the manuscript. We hope that in its present form, our manuscript can be accepted for publication.

Sincerely, on behalf of all co-authors,

Hanneke J.C.M. Wouters, MD
Bruce H.R. Wolffenbuttel, MD, PhD

**Academic Editor:**

1. Please ensure that your manuscript meets PLOS ONE's style requirements, including those for file naming. The PLOS ONE style templates can be found at <https://journals.plos.org/plosone/s/file?id=wjVg/PLOSOne_formatting_sample_main_body.pdf> and <https://journals.plos.org/plosone/s/file?id=ba62/PLOSOne_formatting_sample_title_authors_affiliations.pdf>

We rechecked the style requirements and adapted the manuscript accordingly.

2. We note that Supporting Information file may include questionnaire items that may have been previously published in English. The reproduction of previously published work has implications for the copyright that may apply to these publications. We would be grateful if you could clarify whether you have obtained permission from the original copyright holder to republish these items under a CC BY license. If you have not obtained permission to publish these items please remove them from your manuscript. You may wish to replace the text you have removed with relevant question numbers/ brief descriptions of each item; please be sure to include any relevant references and in-text citations.

In the Supporting Information 3 questionnaire items are described:

- HEALTH73: another disorder that you have not mentioned yet? (baseline)

- HEALTH74: previous thyroid surgery (baseline)

- HEALTH100: another disorder that you have not mentioned yet? (follow-up)

To the best of our knowledge, these questions were formulated by Lifelines and are not part of a previously published questionnaire. The general outline of the Lifelines dataset is publicly available on:

<https://www.lifelines.nl/researcher/data-and-biobank/$6102/$6104>

We have added this to the text.

3. We note that you have indicated that data from this study are available upon request. PLOS only allows data to be available upon request if there are legal or ethical restrictions on sharing data publicly. For information on unacceptable data access restrictions, please see http://journals.plos.org/plosone/s/data-availability#loc-unacceptable-data-access-restrictions.

In your revised cover letter, please address the following prompts:

a) If there are ethical or legal restrictions on sharing a de-identified data set, please explain them in detail (e.g., data contain potentially identifying or sensitive patient information) and who has imposed them (e.g., an ethics committee). Please also provide contact information for a data access committee, ethics committee, or other institutional body to which data requests may be sent.

We will update your Data Availability statement on your behalf to reflect the information you provide.

The results presented in the manuscript are based on the baseline and follow-up measurement of the Lifelines Cohort dataset. Lifelines is the sole owner of any and all data and materials that has been collected from the participants. All data are stored and administered by Lifelines. The data are accessible to researchers through a virtual environment. Data is not publicly available due to restrictions imposed by the Lifelines Scientific Board and the Medical Ethical Committee of the University Medical Center Groningen related to protecting patient privacy and because the participants have not given permission for this. Lifelines is a facility which allows data access for reproducibility of the study results. Questions regarding the data availability can be addressed to the Research Office of Lifelines, [data@research@lifelines.nl](mailto:data@research@lifelines.nl). We have added this information to the data availability statement.

4. We note that you have included the phrase “data not shown” in your manuscript. Unfortunately, this does not meet our data sharing requirements. PLOS does not permit references to inaccessible data. We require that authors provide all relevant data within the paper, Supporting Information files, or in an acceptable, public repository. Please add a citation to support this phrase or upload the data that corresponds with these findings to a stable repository (such as Figshare or Dryad) and provide and URLs, DOIs, or accession numbers that may be used to access these data. Or, if the data are not a core part of the research being presented in your study, we ask that you remove the phrase that refers to these data.

We have removed this phrase as it is not a core part of our analyses.

**Reviewer #1:**

The manuscript PONE-D-20-13620 is based on an analysis with the Lifelines Cohort Study from the North East region of the Netherlands.

We cordially thank the reviewer for his / her comments.

The geographical feature should be part of the title; example, "Epidemiology of thyroid disorders in the Lifelines Cohort Study (Netherland)"

The authors would like to thank the reviewer for this comment. We agree that the geographical area should be part of the title of the current manuscript. We changed the title to ‘Epidemiology of thyroid disorders in the Lifelines Cohort Study (the Netherlands)’.

Some comments should be included in the abstract (results section) on the results of the FT4 and FT3 levels. Since in the abstract (methods section) it is said “Data on baseline thyroid hormones measurements (TSH, FT4 and FT3) were available in a subset of 39935 participants”.

We thank the reviewer for noting this. In the revised version we mention that over 98% of subjects with TSH between 4 and 10 mUI/L had normal FT4.

Figure 1 is not relevant for publication; your data is included several times in the text.

We have removed Figure 1.

In figure 2, on the Y axis, it should say "Percentage using Levothyroxine" instead of "thyroid hormones", as its title describes.

We thank the reviewer for noting this. Accordingly, we corrected this in the revised version of the figure.

Figure 3 does not include information on statistical analysis (model, correlation, p value ...), and a comment on this would be appropriate.

We added information about the statistical analysis in the legend of the figure.

In Table 4, TSH units of measure are missing. In the last row the total number of participants with treatment is expressed and according to gender, it would be convenient to indicate “total”.

We have corrected this.

In table S2 the P-value should be indicated in a similar way to table S1.

We have corrected this.

The discussion should be structured, to improve its analysis, in the four objectives indicated in the abstract: a) prevalence of thyroid disorders, b) thyroid medication use, c) thyroid hormone levels, and d) validity of thyroid data obtained.

We thank the reviewer for this valuable comment. We now used the following headings to structure the discussion:
A) prevalence of thyroid disorders based on use of thyroid medication
B) prevalence and incidence of thyroid disorders based on thyroid hormone levels
C) prevalence of abnormal TSH levels in individuals using levothyroxine
D) validity of the obtained thyroid data.

The discussion indicates “Our study has several strengths and weaknesses”. Part of these comments and others made in other sections of the manuscript should be included in a new section of the discussion on the limitations of the study.

We started a new section to describe the strengths and limitations of our study. In this section we referred to comments which were made previously in the text.

References 28 and 38 of the manuscript must be completed with volume and / or pages of the publication; 30 (6): 821-828 and 9 (3): 792, respectively.

We have corrected this.

**Reviewer #2:**

The study of Wouters et al. describes the epidemiology of thyroid disorders in the Lifelines Cohort Study. It aims at describing prevalence of thyroid disorders, thyroid medication use and thyroid hormone concentrations in the cohort. The manuscript is clear and concise, results are well described, also figures and tables. I have few comments.

We cordially thank the reviewer for his / her comments.

- Line 179 : a reference should be added to ascertain the fact that the general Dutch population is iodine sufficient

We added the following reference: ‘Verkaik-Kloosterman J, Buurma-Rethans EJ, Dekkers AL, van Rossum CT. Decreased, but still sufficient, iodine intake of children and adults in the Netherlands. Br J Nutr. 2017;117(7):1020-1031.’

- Line 179: anti-thyroid peroxidase Ab were not available: this sentence should be commented in method or discussion section. It is not a limitation since the cohort is large enough but precision should be noted.

We have added a sentence on the usefulness of measuring anti-TPO antibodies in the discussion.

- Line 189: unit is missing after fasting glucose level.

We have corrected this.

- Line 210: source of FT4 and FT3 reference interval should be added.

We added that the references interval was based on local laboratory references values.

- Line 278: Participants using levothyroxine had higher FT4 and lower FT3. Time of blood sample is mentioned in method section, but it is not mentioned whether subjects took levothyroxine before or after blood sample. This result is not discussed in discussion and may be not necessary in the manuscript.

We agree with the reviewer that describing the difference in thyroid hormone levels between thyroid hormone users and non-users is not the goal of this study, but we consider this important information, also in the light of the last remark about difficulties GP’s may have to adjust levothyroxine medication. Also, the relevance of this has been recently illustrated by:

Gullo et al. Levothyroxine monotherapy cannot guarantee euthyroidism in all athyreotic patients. PLoS One 2011;6(8):e22552.

and

Flinterman et al. Impact of a forced dose-equivalent levothyroxine brand switch on plasma thyrotropin: a cohort study. Thyroid 2020;30(6):821-828.

We have added this to the discussion.

- Line 286: Figure 3 could be improved by mentioning on it the 98.4% of subjects with normal FT4 and TSH between 4 and 10mUI/L.

We added in the legend of the figure that 98.4% of the individuals with a TSH between 4.01 and 10.0 mU/l had a normal FT4 (>11 pmol/l).

- Line 413: a study in France has been cited mentioning the need of age and sex specific reference values for TSH. Authors could have discussed this sentence in regard of their data: they mentioned that FT4 is normal in 98.4% of subjects with TSH between 4.0 and 10 mUI/L and that less than 60% of subject using levothyroxine have normal TSH. Also G.P. may have didifficulties to adjust levothyroxine medication.

We thank the reviewer for this valuable comment. The increasing upper limit of normal of TSH with aging, as presented in the study of Raverot et al., might partly explain the large number of individuals with a normal T4 in combination with a slightly elevated TSH as well as the large number of individuals treated with levothyroxine having an abnormal TSH. We extended the discussion section commenting on this.
