# Supplementary material for: Epidemiology of thyroid disorders in the Lifelines Cohort Study (the Netherlands)
Source: PLoS One. 2020 Nov 25;15(11):e0242795. doi: 10.1371/journal.pone.0242795 (PMC7688129; doi:10.1371/journal.pone.0242795)
Supplement: Attachment — Submitted filename: Response to Reviewers.docx [file pone.0242795.s007.docx]

P.O. Box 30 001, 9700 RB Groningen, the Netherlands

**Internal Medicine**

Head of department	Prof. dr. R.O.B. Gans

Division	Endocrinology

Head of Division	Prof. dr. B.H.R. Wolffenbuttel

To:

PLOS ONE
Prof. J. Heber and Dr. Naitza
Editor-in-Chief and Academic Editor

Date: November 5, 2020

Phone	0031 50 361 3962
E-mail	bwo@umcg.nl

Dear Professor Heber and Dr. Naitza,

We would like to thank you for your decision dated 27-10-2020 and for another opportunity to resubmit a revised version of our manuscript entitled ‘Epidemiology of thyroid disorders in the Lifelines Cohort Study (the Netherlands)’ (PONE-D-20-13620R1).

In the revised manuscript, we have considered the comments outlined by the reviewers and have modified the manuscript accordingly. The comments are outlined below, followed by our response in a point-by-point manner.

We hope that in its present form, our manuscript can be accepted for publication.

Sincerely, on behalf of all co-authors,

Hanneke J.C.M. Wouters, MD
Bruce H.R. Wolffenbuttel, MD, PhD

**Reviewer #1:**

Thank you for addressing my comments and for making edits to your paper. However, in the manuscript of version R1, figures 2 and 3 modified according to the indications of the text are not included.

We thank the reviewer for his / her comment. In the submitted revised version of the manuscript we included 2 figures (‘Fig 1. Percentage of levothyroxine users per age group’ and ‘Fig 2. Relationship between TSH and FT4 in participants with subclinical or overt hypothyroidism not using levothyroxine’). As suggested we changed the Y axis label into ‘Percentage using Levothyroxine’ in Fig. 1 and added information about the statistical analysis in the legend of Fig 2.

In the PDF proof, which we received during the submission process, the figures are on page 45 and 46.

We hope that in this resubmission the figures are correctly shown and that we have informed you sufficiently.

**Reviewer #2:**

All comments have been adressed. The revised manuscript is clear. The correct unit for TSH is mUI/L, this should be corrected.

We thank the reviewer for noting this. Accordingly, we corrected this in the revised version of the text and in Figure 2.
